# Supplementary material for: Simultaneous Extraction of Bioactive Compounds from Olea europaea L. Leaves and Healthy Seed Oils Using Pressurized Propane
Source: Foods. 2023 Feb 23;12(5):948. doi: 10.3390/foods12050948 (PMC10000711; doi:10.3390/foods12050948)
Supplement: Supplementary file 1 [file foods-12-00948-s001.zip › foods-2163733-supplementary.pdf]

Table S1. Correlation matrix (Pearson) between different evaluated compounds.

Variables	Lauric	Myristic	Palmitic	Palmitoleic	Stearic	Oleic	Linoleic	Linolenic	Arachidic	Gondoic	Behenic	Lignoceric	1-Octacosanol	γ-Tocopherol	α-Tocopherol	Fagarol	Campesterol	Stigmasterol	1-Triacontanol	β-Sitosterol	Pregn-5-en-3-ol	Yield	DPPH	Induction time
Lauric	**1**	0.534	-0.851	**-0.999**	-0.750	-0.775	-0.770	0.775	-0.852	**-0.981**	-0.907	0.123	-0.346	0.285	-0.403	-0.713	-0.718	-0.895	-0.426	-0.365	-0.774	0.142	0.852	-0.724
Myristic	0.534	**1**	-0.843	-0.513	-0.903	-0.885	-0.884	0.883	-0.800	-0.665	-0.368	0.812	0.565	0.710	0.549	**-0.973**	**-0.971**	-0.431	0.517	0.576	-0.839	-0.715	0.344	-0.647
Palmitic	-0.851	-0.843	**1**	0.849	**0.985**	**0.991**	**0.990**	**-0.991**	**0.996**	0.937	0.810	-0.625	-0.036	-0.369	-0.036	0.922	0.941	0.848	0.010	-0.062	**0.988**	0.227	-0.793	0.914
Palmitoleic	**-0.999**	-0.513	0.849	**1**	0.747	0.772	0.768	-0.773	0.855	**0.980**	0.926	-0.120	0.381	-0.238	0.431	0.694	0.702	0.914	0.456	0.396	0.777	-0.179	-0.876	0.742
Stearic	-0.750	-0.903	**0.985**	0.747	**1**	**0.999**	**0.999**	**-0.999**	**0.977**	0.864	0.714	-0.748	-0.180	-0.404	-0.194	0.943	**0.963**	0.766	-0.144	-0.215	**0.991**	0.356	-0.708	0.905
Oleic	-0.775	-0.885	**0.991**	0.772	**0.999**	**1**	**1.000**	**-1.000**	**0.985**	0.883	0.745	-0.723	-0.135	-0.379	-0.149	0.934	**0.955**	0.795	-0.099	-0.170	**0.995**	0.314	-0.738	0.917
Linoleic	-0.770	-0.884	**0.990**	0.768	**0.999**	**1.000**	**1**	**-1.000**	**0.985**	0.880	0.744	-0.728	-0.136	-0.373	-0.151	0.932	**0.953**	0.794	-0.101	-0.172	**0.995**	0.314	-0.738	0.920
Linolenic	0.775	0.883	**-0.991**	-0.773	**-0.999**	**-1.000**	**-1.000**	**1**	**-0.986**	-0.883	-0.747	0.722	0.132	0.376	0.146	-0.933	**-0.954**	-0.796	0.096	0.167	**-0.995**	-0.311	0.741	-0.919
Arachidic	-0.852	-0.800	**0.996**	0.855	**0.977**	**0.985**	**0.985**	**-0.986**	**1**	0.937	0.848	-0.613	0.033	-0.282	0.022	0.884	0.908	0.885	0.072	0.000	**0.991**	0.154	-0.839	0.945
Gondoic	**-0.981**	-0.665	0.937	**0.980**	0.864	0.883	0.880	-0.883	0.937	**1**	0.907	-0.314	0.222	-0.318	0.259	0.813	0.824	0.913	0.292	0.225	0.882	-0.016	-0.865	0.824
Behenic	-0.907	-0.368	0.810	0.926	0.714	0.745	0.744	-0.747	0.848	0.907	**1**	-0.170	0.557	0.110	0.547	0.539	0.572	**0.996**	0.590	0.530	0.786	-0.387	**-0.993**	0.860
Lignoceric	0.123	0.812	-0.625	-0.120	-0.748	-0.723	-0.728	0.722	-0.613	-0.314	-0.170	**1**	0.604	0.302	0.685	-0.693	-0.719	-0.260	0.633	0.678	-0.713	-0.662	0.217	-0.640
1-Octacosanol	-0.346	0.565	-0.036	0.381	-0.180	-0.135	-0.136	0.132	0.033	0.222	0.557	0.604	**1**	0.698	**0.985**	-0.383	-0.356	0.494	**0.992**	**0.992**	-0.061	**-0.978**	-0.566	0.166
γ-Tocopherol	0.285	0.710	-0.369	-0.238	-0.404	-0.379	-0.373	0.376	-0.282	-0.318	0.110	0.302	0.698	**1**	0.580	-0.685	-0.635	0.090	0.603	0.625	-0.284	-0.802	-0.196	0.021
α-Tocopherol	-0.403	0.549	-0.036	0.431	-0.194	-0.149	-0.151	0.146	0.022	0.259	0.547	0.685	**0.985**	0.580	**1**	-0.349	-0.332	0.475	**0.997**	**0.998**	-0.088	-0.949	-0.535	0.102
Fagarol	-0.713	**-0.973**	0.922	0.694	0.943	0.934	0.932	-0.933	0.884	0.813	0.539	-0.693	-0.383	-0.685	-0.349	**1**	**0.998**	0.586	-0.319	-0.384	0.893	0.563	-0.501	0.714
Campesterol	-0.718	**-0.971**	0.941	0.702	**0.963**	**0.955**	**0.953**	**-0.954**	0.908	0.824	0.572	-0.719	-0.356	-0.635	-0.332	**0.998**	**1**	0.622	-0.298	-0.364	0.921	0.535	-0.541	0.758
Stigmasterol	-0.895	-0.431	0.848	0.914	0.766	0.795	0.794	-0.796	0.885	0.913	**0.996**	-0.260	0.494	0.090	0.475	0.586	0.622	**1**	0.522	0.460	0.835	-0.323	**-0.994**	0.904
1-Triacontanol	-0.426	0.517	0.010	0.456	-0.144	-0.099	-0.101	0.096	0.072	0.292	0.590	0.633	**0.992**	0.603	**0.997**	-0.319	-0.298	0.522	**1**	**0.997**	-0.034	**-0.951**	-0.584	0.165
β-Sitosterol	-0.365	0.576	-0.062	0.396	-0.215	-0.170	-0.172	0.167	0.000	0.225	0.530	0.678	**0.992**	0.625	**0.998**	-0.384	-0.364	0.460	**0.997**	**1**	-0.105	**-0.965**	-0.525	0.097
Pregn-5-en-3-ol	-0.774	-0.839	**0.988**	0.777	**0.991**	**0.995**	**0.995**	**-0.995**	**0.991**	0.882	0.786	-0.713	-0.061	-0.284	-0.088	0.893	0.921	0.835	-0.034	-0.105	**1**	0.236	-0.788	**0.953**
Yield	0.142	-0.715	0.227	-0.179	0.356	0.314	0.314	-0.311	0.154	-0.016	-0.387	-0.662	**-0.978**	-0.802	-0.949	0.563	0.535	-0.323	**-0.951**	**-0.965**	0.236	**1**	0.408	-0.015
DPPH	0.852	0.344	-0.793	-0.876	-0.708	-0.738	-0.738	0.741	-0.839	-0.865	**-0.993**	0.217	-0.566	-0.196	-0.535	-0.501	-0.541	**-0.994**	-0.584	-0.525	-0.788	0.408	**1**	-0.889
Induction time	-0.724	-0.647	0.914	0.742	0.905	0.917	0.920	-0.919	0.945	0.824	0.860	-0.640	0.166	0.021	0.102	0.714	0.758	0.904	0.165	0.097	**0.953**	-0.015	-0.889	**1**

Values in bold are different from 0 with a significance level α=0.05.
